# Supplementary material for: Peroxisome Proliferator-Activated Receptor-α (PPARα) Expression in a Clinical Population of Pakistani Patients with Type 2 Diabetes and Dyslipidemia
Source: Int J Mol Sci. 2022 Sep 16;23(18):10847. doi: 10.3390/ijms231810847 (PMC9505490; doi:10.3390/ijms231810847)
Supplement: Supplementary file 1 [file ijms-23-10847-s001.zip › ijms-1868089-supplementary.pdf]

Supplemented data:

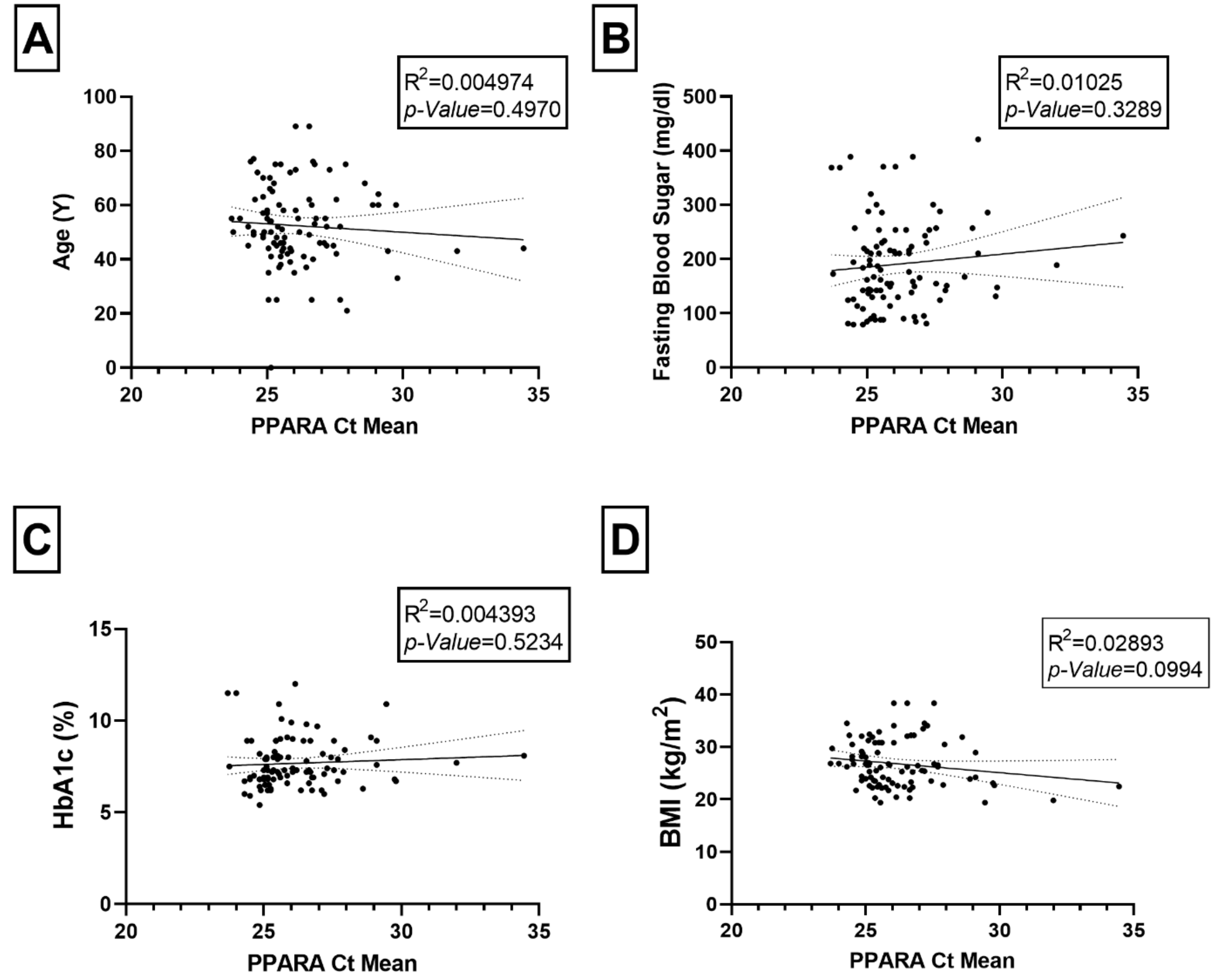

Figure S1: Correlation analysis between PPARα gene expression and different T2DM Parameters.

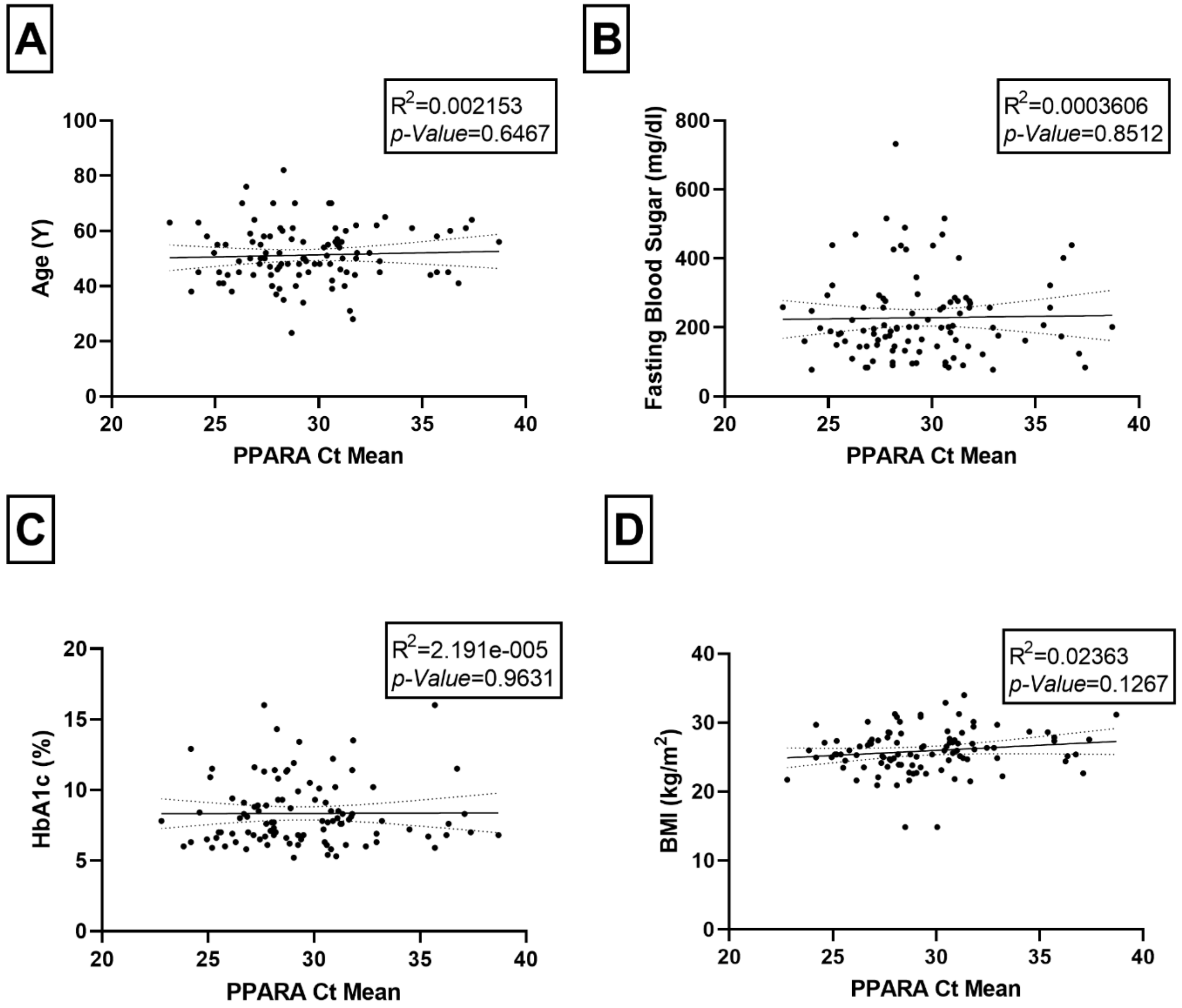

Figure S2: Correlation analysis between *PPARa* gene expression and different DD Parameters.

Supplementary Table S1: Diagnostic criteria for normal levels of lipid profile.

Sr No.	Parameters	Normal Ranges	
		Male	Female
1	Total cholesterol	125-200 mg/dl<br>3.23- 5.17mmol/L	125-200 mg/dl<br>3.23- 5.17mmol/L
2	Triglycerides	130 mg/dl<br>1.47 mmol/L	130 mg/dl<br>1.47 mmol/L
3	Low-density lipoprotein-cholesterol	<100 mg/dl<br><2.58 mmol/L	<100 mg/dl<br><2.58 mmol/L
4	High-density lipoprotein-cholesterol	>50 mg/dl<br>>1.0 mmol/L	>40 mg/dl<br>>1.0 mmol/L

Supplementary Table S2: Diagnostic criteria for type 2 diabetes.

**Sr No.**	**Parameters**	**Normal Ranges**	
		**Male**	**Female**
**1**	Fasting blood glucose	>7.0 mmol/L<br>126mg/dl	>7.0 mmol/L<br>126mg/dl
**2**	2 hours post-load plasma glucose	11.1 mmol/L<br>200 mg/dl	11.1 mmol/L<br>200 mg/dl
**3**	Random plasma glucose	11.1 mmol/L<br>200 mg/dl	11.1 mmol/L<br>200 mg/dl
**4**	HbA1c	6.5%	6.5%

Supplementary Table S3: Gender-adjusted regression analysis of PPARα expression.

	p-values of the control group (n=100) after regression analysis with PPARα expression		*p-values* of T2DM group (n=100) after regression analysis with PPARα expression.		*p-values* of DD group (n=100) after regression analysis with PPARα expression.	
	Male	Female	Male	Female	Male	Female
Age	0.666	0.972	0.775	0.306	0.256	0.153
HbA1c (mmol/mol)	0.004	0.445	0.125	0.937	0.295	0.474

Fasting blood glucose (mg/dl)	0.035	0.780	0.016	0.841	0.766	0.861
tchol (mg/dl)	0.472	0.461	0.28	0.96	0.593	0.875
TAGS (mg/dl)	0.383	0.427	0.491	0.547	0.054	0.872
LDL (mg/dl)	0.396	0.225	0.586	0.592	0.724	0.323
HDL (mg/dl)	0.108	0.822	0.953	0.289	0.756	0.718
